# How (not) to Talk to a Plant: An Application of Automata Theory to Plant Communication

**DOI:** 10.1007/s10441-024-09484-y

**Published:** 2024-06-29

**Authors:** Lorenzo Baravalle

**Affiliations:** Centro de Filosofia das Ciências, Departamento de História e Filosofia das Ciências, Faculdade de Ciências, Universidade de Lisboa Campo Grande, Edifício C4, 3º Piso, Sala 4.3.24, 1749-016 Lisbon, Portugal

**Keywords:** Plant cognition, Plant communication, Chomsky’s hierarchy, Automata theory, Comparative approach

## Abstract

Plants are capable of a range of complex interactions with the environment. Over the last decade, some authors have used this as evidence to argue that plants are cognitive agents. While there is no consensus on this view, it is certainly interesting to approach the debate from a comparative perspective, trying to understand whether different lineages of plants show different degrees of responsiveness to environmental cues, and how their responses compare with those of animals or humans. In this paper, I suggest that a potentially fruitful approach to these comparative studies is provided by automata theory. Accordingly, I shall present a possible application of this theory to plant communication. Two tentative results will emerge. First, that different lineages may exhibit different levels of complexity in response to similar stimuli. Second, that current evidence does not allow to infer great cognitive sophistication in plants.

## Introduction

While plants are sometimes considered as stereotyped organisms, incapable of the rich interactions with the environment that characterise animals, plant biology delivers us a different picture. Plants can perform a wide range of complex behaviours, related to their growth, development and maintenance, such as a variety of tropic movements (e.g., phototropism, chemotropism, thermotropism, etc.), nastic movements (i.e., non-directional responses to external stimuli), or even locomotion (for instance, in certain motile algae). This has led some authors (e.g., Trewavas 2015; Mancuso 2018; Gagliano 2018; Novoplansky 2019; Calvo 2023) to argue that plants are autonomous cognitive agents. Rather than merely reacting to environmental conditions, plants are seen by these authors as capable of a variety of intelligent or learning-informed actions, such as exploration, guessing or prediction. But even if we accept these cognitivist reconstructions of plant behaviour, it is natural to wonder how plant intelligence compares with that of animals or humans. After all, if we discover that plants’ cognitive abilities are substantially limited by some–possibly non-anthropocentric (but rather “biogenic”; Lyon 2006)–standards, then we would have good reasons to take a more cautious or even sceptical view (e.g., Adams 2018, Taiz et al. 2019; Mallat et al. 2020). A crucial question at this juncture is: is it possible to measure the cognitive sophistication of a plant?

Cognition is generally understood as the ability to generate “flexible goal-oriented behavior through information processing” (Andrews and Monsó 2021, p. 3). Accordingly, we can define “cognitive sophistication” as the degree of flexibility involved in a goal-oriented behaviour. Clarifying the notion of “flexibility” involved in this definition is one of the aims of this article. When considering animals, common criteria for assessing their cognitive abilities and sophistication include whether they exhibit problem-solving abilities such as object manipulation (Parker and Gibson 1990; Osvath and Osvath 2008), spatial reasoning (Gibson et al. 2011) or tool use (Boesch 1991), social cognition involving empathic responses (Preston and de Waal 2004), a theory of mind (Penn and Povinelli 2007) or social learning (Whiten et al. 2009), and self-awareness (in terms of self-recognition or self-agency; e.g., Gallup 1998). Many of these criteria are inappropriate for assessing plant cognition, either because they are too “zoocentric” (e.g., tool use or a theory of mind) or because they are difficult or impossible to detect experimentally in plants (e.g., self-awareness).

Communication has been extensively studied in animals since Bradbury and Vehrencamp (1998) and Maynard-Smith and Harper (2003), and has also attracted the attention of many plant biologists (e.g., Gagliano 2012; Karban 2021) and linguists (Bonato et al. 2021) as a proxy for identifying cognitive abilities in plants. As a field, plant communication encompasses at least two broad categories of phenomena. On the one hand, we have natural communication between plants, mediated chemically or acoustically. On the other hand, we have artificial communication between human experimenters and plants, usually achieved by electrical stimulation, which is increasingly used to implement sustainable biotechnologies (e.g., Li et al. 2021; Garcia-Servín et al. 2021). Although the ultimate goal of this interaction is to gain control over the plant’s behaviour, it can still be considered a form of communication in that this goal is achieved by establishing protocols for the transmission and exchange of information between the human experimenter and the plant.

In Sect. 2, I shall provide a brief overview of both lines of research. Although I shall focus on the latter in the remainder of the article, the approach developed here might be profitably applied to analyse plant-plant communication as well. In Sect. 3, I shall present my proposal, i.e., to use Chomsky hierarchy and automata theory as tools for assessing the cognitive sophistication involved in plant’s communication. Chomsky’s hierarchy was originally introduced by Chomsky (1959) to identify differences between the generative power of different types of grammars; computer scientists later proved that the categories of Chomsky hierarchy are formally equivalent to different classes of automata, allowing for a rigorous account of the differences between their computational power and complexity (Kozen 1997; Hopcroft et al. 2008; Sipser 2020). More recently, formal linguists (Jäger and Rogers 2012; Fitch and Friederici 2012; Fitch 2014) have used it to comparatively assess the cognitive resources required to perform specific communicative interactions, both in humans and other animals. In these applications, responsiveness to different grammars serves as a comparative measure of an organism’s behavioural flexibility, which in turn is assumed to provide information about its cognitive sophistication.

In Sect. 4, I shall interpret the evidence about artificial interactions with plants capable of rapid responses, such as *Dionaea muscipula* (Venus flytrap), *Mimosa pudica*, or plants belonging to the genera *Aldrovanda* and *Drosera*, using the conceptual tools discussed in the previous section. Two main tentative results emerge from my analysis, which I shall discuss in Sect. 5. First, given similar physiological and experimental setups, *Dionaea muscipula* seems to outperform other lineages*,* in the sense that it manages to process external, artificially generated cues which are more complex according to Chomsky’s hierarchy. This is interesting because it suggests that if we adopt Chomsky’s hierarchy as a systematic measure of cognitive complexity, we may be able to identify differences between the cognitive abilities of different plant species (whereas it is usually tacitly assumed that most plants have roughly the same level of cognitive sophistication). A second, less encouraging result–at least for the proponents of plant intelligence–is that *Dionaea muscipula*, despite outperforming other lineages (at least in the specific communicative task I shall consider in this article), shows a very reduced behavioural flexibility and thus–according to our assumptions–limited cognitive complexity. Of course, it may be that other species engaged in other communicative interactions exhibit more complex behaviour; accordingly, I shall suggest some possible directions for further research.

## Plant communication

Plant communication is a rapidly expanding field. Despite a false start in the 1970s–often attributed to the publication of Tompkins and Bird’s (in)famous The Secret Life of Plants (1973) and the subsequent controversies surrounding it–more solid evidence for the ability of plants to communicate began to emerge in the 1990s. In the words of Karban (2021), “communication occurs when a sender emits a cue perceived by a receiver that changes the receiver’s behaviour” (p. 1). This definition, implicitly accepted by most researchers, allows for the inclusion of both “true” communication, which is achieved when the sender intentionally chooses to emit a signal, and eavesdropping, where an unintentionally emitted cue is exploited by the receiver (Bradbury and Vehrencamp 1998; Maynard-Smith and Harper 2003). Notwithstanding other possible interpretations, the intentional/unintentional distinction in this characterisation allows for a naturalistic elucidation as follows. Plants, like any other organism, live in populations of related individuals. According to kin selection theory (Hamilton 1964; Birch 2017), it is adaptive for many species to evolve behaviours that increase the reproductive chances of relatives in specific ecological circumstances. Under this widely accepted assumption, some plant species (e.g., sagebrush, *Artemisia tridentata*; Karban et al. 2006, 2013), might have evolved mechanisms to emit cues (mainly volatile organic compounds; VOCs) to signal the presence of competitors or attackers to their relatives. Communicative interactions resulting from the evolution of such mechanisms would be “intentional” as opposed to “unintentional” eavesdropping, where the behaviour of the sender has not been selected.^1^

Recent studies on plant-plant communication have focused on two main channels of plant communication, namely VOCs and acoustic vibrations (AVs). VOCs can be emitted by roots to communicate the presence of water or mineral resources (Kong et al. 2018), or to promote symbiotic responses with fungi (Oldroyd 2013). However, the most studied type of VOCs are probably airborne VOCs, such as terpenoids (Peñuelas et al. 1995; Peñuelas and Llusià 2004). Airborne VOCs are emitted by plants to attract pollinators (Slavković and Bendahmane 2023), to communicate danger and elicit defence (e.g., in tomato plants; Sugimoto et al. 2014), or to attract predators against their attackers (e.g., maize seedlings attract wasps that parasitise the caterpillars that attack them). Bonato et al. (2021) argues that terpenoids have a molecular structure with combinatorial features that can be interpreted as endowing them with syntactical and semantic properties. Although they do not explicitly present the linguistic features of terpenoids in detail, they seem to suggest that plant communication may have the same degree of complexity as animal communication: for example, it is sensitive to pragmatic, contextual, conditions and displays dialectic variants (like birdsongs).

AVs have only more recently been taken into account, mainly thanks to the work of Chamovitz (2012), Gagliano (2012; Gagliano et al. 2012) and Appel and Cocroft (2014). According to Gagliano (2012), plants both produce and perceive sound. Acoustic emissions are usually produced by the release of tension in xylemic water transport systems, although Gagliano et al. (2012) believe that they can be produced independently of hydraulic processes. The vibrations of motor proteins such as myosin could be used to produce specific sounds. Regardless of the physicochemical origins of plant sound emission, Gagliano believes that AVs can perform similar functions to those of VOCs. Mishra et al. (2016) investigates the physiological mechanisms responsible for the perception of AVs in plants. They also discuss the implications of this research for biotechnology and agriculture. Sound treatments have been used in a variety of plantations, particularly in China, to improve production. Plants treated with AVs display increased immune responses against diseases and insect pests (see also Hassanien et al. 2014).

The commercial relevance of plant communication has stimulated another strand of research, that of artificial communication. To improve biotechnology through plant communication, researchers need to find the best way to elicit the desired responses. While VOCs and AVs are thought to be natural channels for plant-plant communication, we do not yet know enough about how they work. Nevertheless, there is another class of cues that can be manipulated more efficiently: electric signals (ESs). García-Servín et al. (2021) review different types of ESs in plants, that is, action potentials (APs), variation potentials (VPs), and system potentials (SPs). APs are the most widely studied and used to elicit specific responses in plants. This is because APs are typically generated by non-critical damage and, therefore, can be manipulated to convey information without causing possible disruptions (Sukhova et al. 2017). Moreover, APs consist of sequences of single and fast spikes, followed by short periods of depolarisation and repolarisation. This, as we shall see in more detail in Sect. 4, makes them ideal candidates for encoding digital information.

A good example of artificial communication is provided by Li et al.’s (2021) experiments with soft actuators. Soft actuators (also called *phytoactuators*; Volkov and Markin 2012) are non-invasive electrodes that adapt to the surface of a plant. These are positioned on the surface of the lobes of a Venus flytrap’s (*Dionaea muscipula*). The electrodes both detect ESs from the plant and deliver APs to it. A communication channel is then established via a Wi-Fi connection between the actuator and a mobile application. By electrically stimulating the plant, the researchers are able to open and close the lobes on demand and make the plant grasp small objects. Meder et al. (2023) mention a number of other examples and applications of artificial communication, such as the creation of hybrid (plant-machine) devices capable of harvesting energy and used as antennas to power sensors monitoring environmental variables.

## Chomsky and subregular hierarchies

**Fig. 1:**
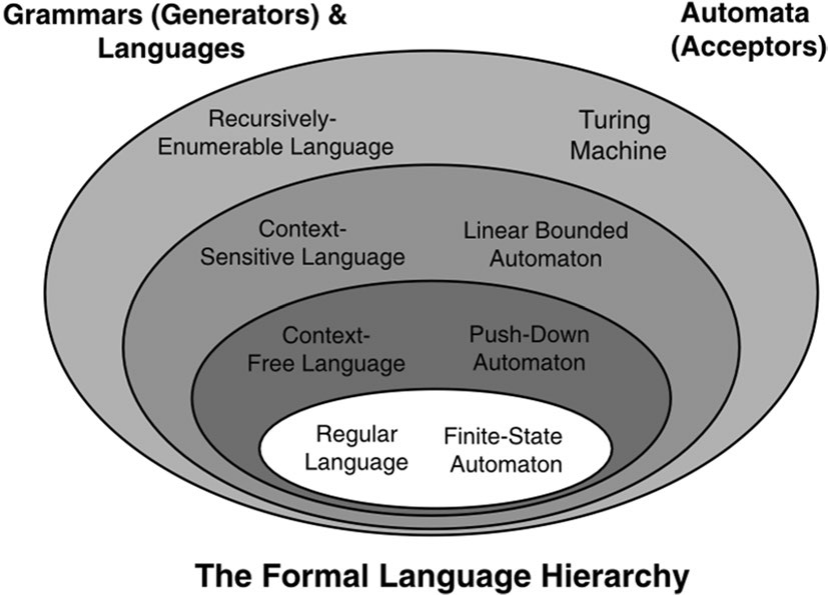
A graphical representation of the equivalence between Chomsky hierarchy and automata

Chomsky hierarchy is usually depicted as in Fig. 1 (from Fitch 2014, p. 348).

There are four strictly nested classes of grammars–from the bottom up, regular grammars, context-free grammars, context-sensitive grammars and recursively-enumerable–which are equivalent to four classes of automata–finite-state automata, push-down automata, linear bounded automata and Turing machines, respectively. In many standard presentations of Chomsky’s hierarchy (e.g., Hopcroft et al. 2008; Sipser 2020), context-sensitive and recursively enumerable grammars are lumped into a single category, equivalent to Turing machines. Since the difference between these grammars is not relevant in the present context, we can follow this convention and simply assume that any grammar that is not context-free but recursively enumerable can generate strings that cannot be accepted by any other kind of automaton except Turing machines.

A grammar can be formally defined as a tuple $\mathcal{G}= <\Sigma , V, S, R>$. $\Sigma$ is the set of symbols that form the strings of the language *L* generated by $\mathcal{G}$, called terminals. V is a set of variables called *nonterminals* (or *syntactic categories*). Note that nonterminals are not symbols of *L*, but rather “placeholders” for symbols of *L*. $S \subseteq V$ is the initial symbol, from which we can generate all the strings of *L*. *R* is a set of *rules* that generate the strings of the language. Rules can be represented as $\alpha \to \beta$, where $\alpha \subseteq V$, $\beta$ is a string that can be formed either of terminals or nonterminals or both, and $\to$ means “replaces with”, or “consists of”. From S and other nonterminals, rules can be applied recursively to obtain strings containing only terminals. Thus, for instance, suppose that we have a grammar ${\mathcal{G}}_{1}$ with $\Sigma =\left\{a, b, c, d\right\}$, $V=\{S, T\}$ and the following rules (Jäger and Rogers 2012, p. 1958):

$$ {\text{I}}{.}\quad \;S \to aTd $$

$$ {\text{II}}{.}\quad \,T \to bSc $$

$$ {\text{III}}.\quad T \to bc $$

Then any string in our language will have the form $aTd$; i.e., it will begin with *a* and end with *d* (rule I). *T* can be replaced by $bc$ (rule III), in which case we obtain the string *abcd*. This string has no nonterminals and is therefore a string of *L(*${\mathcal{G}}_{1})$ (i.e., the language generated by ${\mathcal{G}}_{1}$). Otherwise, we can replace *T* with another $aTd$ (rule II + rule I), to get *abaTdcd*. Since this string still contains nonterminal variables, it is not a string of *L(*${\mathcal{G}}_{1})$. Thus we have to apply rule III or rule II again (and, in the latter case, rule I one more time, and so forth). As result, we will generate an enumerable infinite set corresponding to the language *L* $({\mathcal{G}}_{1})=\left\{abcd, ababcdcd, abababcdcdcd, \dots \right\}=\{{\left(ab\right)}^{n}\bullet {\left(cd\right)}^{n}|n \in {\mathbb{N}}^{+}\}$ (where $\bullet$ is the concatenation symbol, which will be omitted from now on).

${\mathcal{G}}_{1}$ is an example of a context-free grammar. Although simple, ${\mathcal{G}}_{1}$ displays some important features and limitations of context-free grammars. First, they allow something called “centre embedding”, i.e., the possibility of nesting certain linguistic structures (in the case of ${\mathcal{G}}_{1}$, *abcd*) within others. Note that, despite the name, the “embedding” can be produced also in lateral clauses–i.e., by “unpacking” nonterminal variables at the beginning or end of a string (Fitch 2014, p. 352–353; this is not evident in ${\mathcal{G}}_{1}$ due to its simplicity). Second, and in virtue of centre embedding, context-free grammars display an unlimited capacity for symmetric counting. For instance, ${\mathcal{G}}_{1}$ generates sequences of *ab* and *cd* that are of equal length, regardless of the size of the portions. These two features are absent in regular grammars. Regular grammars cannot generate centre embedded sequences and can at most generate symmetric sequences of symbols of fixed length. But even context-free grammars have limitations. Although they are recursive to a certain extent, context-free grammars are not *generally* recursive. Context-free grammars cannot, for instance, generate languages like *L* $({\mathcal{G}}_{2})=\{{\left(ab\right)}^{n}\bullet {\left(cd\right)}^{n}.{\left(ef\right)}^{n}|n \in {\mathbb{N}}^{+}\}$, which requires multiple embeddings.^2^

In the context of comparative formal linguistics, there is a conjecture that most linguistic structures in human languages can be generated by context-free grammars (Miller 1967). This conjecture has been called the “supra-regular hypothesis” by Fitch (2014). From the supra-regular hypothesis, Fitch derives another hypothesis, the “dendrophilia hypothesis”, which states that it is difficult or impossible for non-human organisms to process supra-regular languages.^3^ This hypothesis is not universally accepted. There is evidence that some birdsongs, for instance, exhibit the complexity of context-free languages (Abe and Watanabe 2011; but see Beckers et al. 2012 for a more sceptical view). However, we can tentatively accept Fitch’s hypothesis here because it helps us to limit possible applications of the Chomsky hierarchy to plant behaviour. *If* non-human organisms cannot process supra-regular languages, then they can only process regular languages. Our focus will therefore be on the latter type of language. So far, we have characterised Chomsky’s hierarchy in terms of grammars. Yet, when we talk about whether or not organisms can “process” a language, it is useful to think of Chomsky’s hierarchy in terms of automata. “To process” in this context is to be able to recognise a particular string as such and eventually produce a particular response–in computational jargon, this is also called “to accept” the string. We can therefore say that Chomsky’s hierarchy distinguishes between grammars of different generative power *and*, equivalently, between automata of different processing power (Kozen 1997). Most importantly for the present case, finite-state automata (which are capable of processing regular languages) are incapable of processing context-free languages. To better understand this point, it is useful to characterise the notion of “acceptance” in the context of finite automata.

A finite-state automaton–hereafter, simply “finite automaton”–is usually defined by a quintuple $<Q,\Sigma , \delta ,{q}_{0},F>$, where *Q* is the finite set of states of the automaton, $\Sigma$ is the *alphabet* (which is equal to the set of terminals generated by the grammar accepted by the automaton), $\delta :Q\times\Sigma \to Q$ is the *transition function*, ${q}_{0}\subseteq Q$ is the *initial state*, and $F\subseteq Q$ is the set of *accepting states* (Sipser 2020, p. 35). There are many possible mechanical descriptions of an automaton, which depend, for instance, on whether we consider the string being processed to be read by the automaton all at once and then parsed internally instead of as a sequence inputted symbol by symbol; or whether we consider the automaton to output intermediate responses (like in a Mealy machine; Mealy 1955) instead of just the response resulting from reaching an accepting state. In general, we can think of a finite automaton as a device that, by reading a string, performs a number of transitions, determined by $\delta$, starting from the initial state. If, after reading all the symbols, the automaton is in a state, $q_{i} \subseteq F$ , it accepts the string, otherwise, if $q_{i} \not\subset F$, it rejects it. In mechanical terms, we can think of a computation that ends in an accepting state as a process that produces a proper “reaction” on behalf of the automaton, while a computation that ends in a non-accepting state simply halts. As mentioned above, finite automata correctly process (i.e., terminate in accepting states) strings generated by a regular grammar. When inputted to a finite automaton, strings generated by supra-regular grammars can cause two kinds of behaviour: either they make the automaton halt on a non-accepting state, or they make the automaton incorrectly end on an accepting state.^4^

**Fig. 2:**
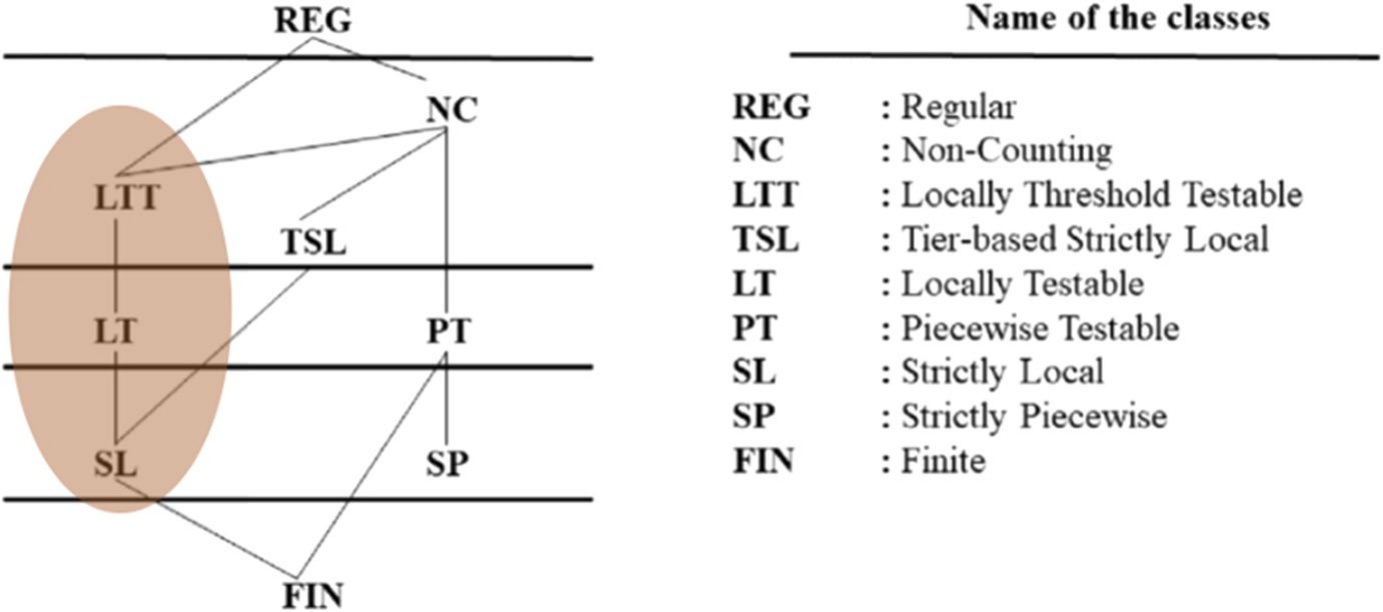
A Hasse diagram of subregular languages (from Avcu and Hestvik 2020). In this article, we shall discuss only the highlighted subset of these languages, corresponding to the automata discussed by Jäger and Rogers (2012)

Before considering applications to plant behaviour, we need to introduce some important classes of automata. Recall that the main goal of this article is to account for differences in the degree of cognitive sophistication in different plant behaviours. Now, *if* the dendrophilia hypothesis is true (as we tentatively assume in this article), then all animals and plants have at most the capacity to process regular languages, i.e., they work like finite automata (we shall discuss in what sense plants “process languages” in the following sections; for the moment, let us just assume that they do). If that were all, then we would have nothing interesting to say about different levels of complexity in plants using Chomsky’s hierarchy. Finite automata are at the bottom of the hierarchy. Therefore, animals and plants are *at best* capable of the most limited processing skills, end of story. Luckily for us, however, Jäger and Rogers (2012; Rogers et al. 2013) have identified some interesting subclasses of regular languages, which display an inner richness within this class of grammars. Jäger and Rogers distinguish three main subregular languages, from the simplest to the most complex, which are generated by corresponding grammars and accepted by specific automata (Fig. 2).

**Fig. 3:**
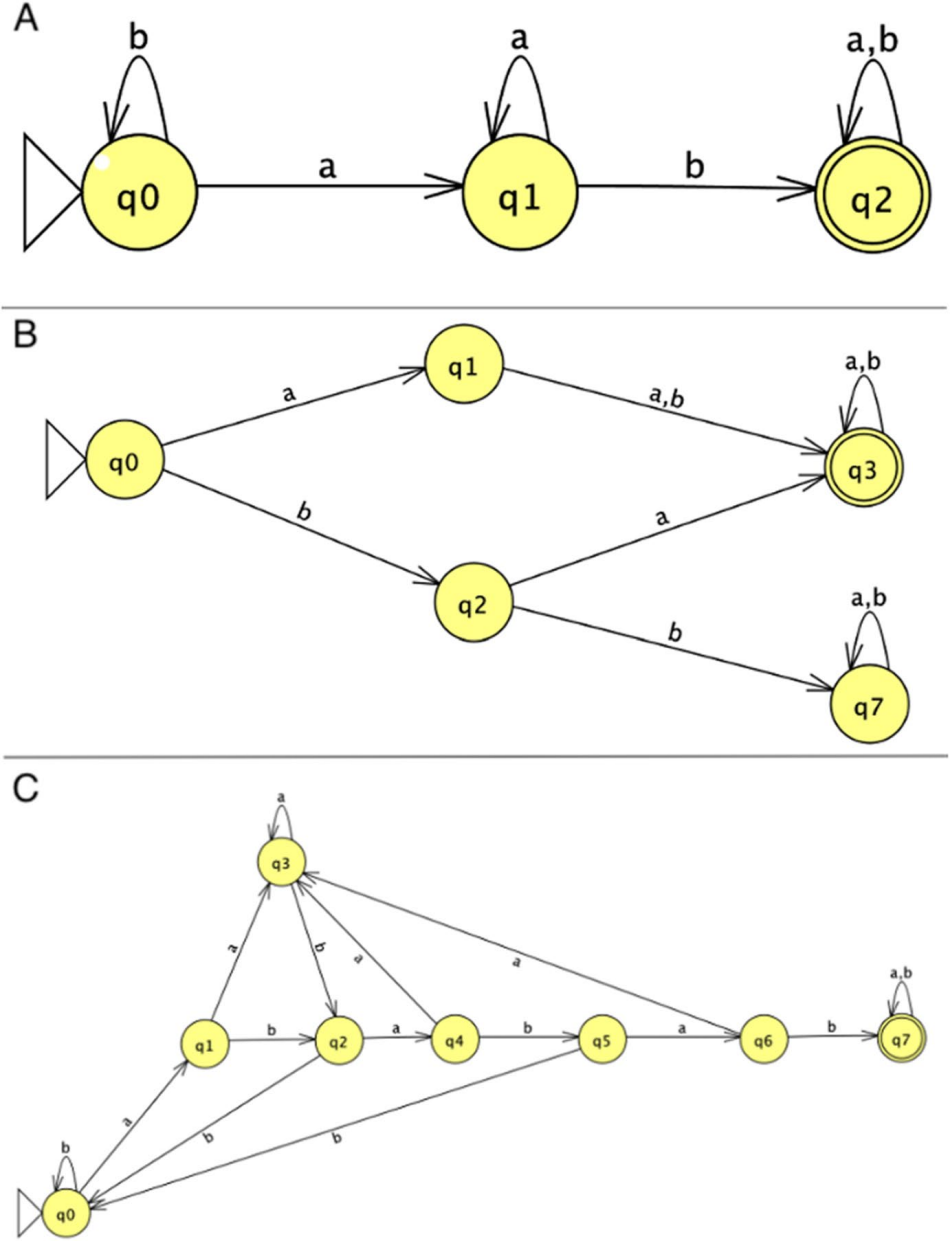
A graphical representation of: **a** a scanner; **b** *L* $({LT}_{1})$ acceptor; **c** *L* $({LTT}_{1})$ acceptor

These are *strictly local* (SL), *locally testable* (LT), and *locally threshold testable* (LTT) *languages*. SL languages are very simple. They are generated to contain a fixed sequence of adjacent symbols *k* (e.g., the 2-sequence “ab”). The corresponding automata, called *scanners* (Fig. 3A), read a string and, if they find the *k*-sequence, accept the string, otherwise they reject the string (or halt without accepting). Scanners are insensitive to any other properties of the string, such as the repetition of *k*-sequences. LT languages are more interesting. The corresponding grammars generate variable sequences of symbols–e.g., a grammar which, given Σ = {a, b}, generates *L* $({LT}_{1})=\{{\left(ab|aa|ba\right)}^{*}\}$ (where the star symbol stands for zero or more repetitions and the pipe symbol for OR).^5^ The automata that accept such languages–let us call them LT-acceptors (Fig. 3B)–must be able to perform some kind of Boolean operation on a possible input. Since they can recognise more than one kind of sequence and have to discard others (in this case “*bb*”), they have to keep track of the last symbols read to check if the next one will satisfy one of the allowed combinations. Thus, for instance, an acceptor of ${LT}_{1}$ would have to “remember” whether the symbol is *a* OR *b AND*, in the latter case, whether the next symbol is *a* and *NOT b*.

Despite having a rudimentary form of memory, LT acceptors, like SL acceptors, are unable to *count* the number of *k*-sequences in a string. They accept if some of the *k*-sequences are found in the string, otherwise they reject or halt. This limitation is overcome by LTT acceptors. LTT languages are languages that have a number of repetitions of a certain patter. Take, for instance, *L* $({LTT}_{1})=\{{\left(aa|bb|ba\right)}^{*}ababab{\left(a|b\right)}^{*}\}$, where Σ = {a, b} as before. *L* $({LTT}_{1})$ contains string of arbitrary length including *at least* three adjacent *ab* sequences. Thus, the corresponding LTT acceptors (Fig. 3C) must be able to quantify portions of the string by recording the number of occurrences of *ab*. If *ab* occurs at least three times, then the LTT acceptor accepts the string. Otherwise, if *ab* occurs less than three times, the LTT acceptor rejects or halts.^6^

I shall say more about subregular automata when, in the next section, I suggest how they can be used to model plant behaviour. For now, just note that while the subregular hierarchy has mainly been used to model phonological elements of human languages (e.g., Avcu and Hestvik 2020), its broader relevance to understanding differences between human and animal cognitive abilities is usually acknowledged (Jäger and Rogers 2012; Fitch and Friederici 2012; more on this in Sect. 5). The requirements for applying Chomsky’s hierarchy (or, more specifically, the subregular hierarchy) to real-world systems are mainly two. First, we need to identify a class of signals that embodies the vocabulary of the strings that can be read by the system being characterised as an automaton. Second, we need to identify behavioural responses that correspond to the outputs of the accepting states of the automaton.

## Plants as automata

Regarding the first of the two requirements mentioned at the end of the previous section, a problem for modelling plant communication in terms of Chomsky’s hierarchy is the limited experimental evidence available. Although there is a considerable amount of research on the subject (see Sect. 2), the details of the communicative interaction are often unknown. ES’ signalling is a partial exception to this state of affairs. Studies such as García-Servín et al. (2021) show that it is possible to identify voltage ranges to which specific plants are sensitive. For example, *Dionaea muscipula* and *Mimosa pudica*, both plants capable of rapid movement, show responsiveness to a voltage range between 0.25 and 2 V. We can therefore tentatively interpret AP sequences as generated from a vocabulary Σ = {0, 1}, where 1 corresponds to an AP with a voltage between 0.25 and 2 V, and 0 to the absence of voltage in this range.

Regarding the second requirement, the choice of fast-moving plants as a case study is clearly due to the fact that we can test their responses in real time. Changes in temperature or light, for instance, cause a variety of responses in different plants, but most of these responses are only detectable in the medium to long term (e.g., they stimulate the release of specific chemicals that affect growth and development, or crop production). Although this type of response could be interpreted according to the approach I will propose, it would be difficult to determine the details of how the plant processes the information (either because the molecular basis of the responses is unknown or because it is impossible to detect when the responses happen as they are gradual). On the other hand, responses to stimulation by VOCs and AVs (Sect. 2) would provide ideal experimental protocols for the kind of model proposed here because of their relatively short duration. But again, the evidence about these processes is still too fragmentary to allow a proper conceptualisation.

Another reason for choosing *Dionaea muscipula* and *Mimosa pudica*, along with plants of the genera *Aldrovanda* and *Drosera*, as model organisms is that they all display similar mechanisms of ES processing (Volkov et al. 2009; Pavlovic et al. 2017; Krausko et al. 2017). Roughly, this processing can be described as follows. First, a specific electrical cue–or sequence of electrical cues–is perceived on the leaves of the plant. This leads to the opening of water channels (called in *Dionaea*, *Aldrovanda* or *Drosera*–which are all carnivorous plants–“plasmodesmata”, and “gap junctions” in *Mimosa*) between hydraulic reservoirs. The opening of the water channels is triggered by an increase in cytosolic calcium concentration. In carnivorous plants, the calcium influx activates calcium-dependent protein kinases, which phosphorylate various proteins involved in the opening of plasmodesmata. In *Mimosa*, the calcium influx activates a mechanosensitive channel protein that allows the influx of potassium ions, which in turn triggers the opening of gap junctions. The opening of the water channels leads to a rapid increase in the flow of water from the storage cells to the motor cells. This increase in water pressure within the motor cells is the primary driving force behind the movement of the trap or leaflets. In *Dionaea*, the water pressure causes the hinge cells to swell, forcing the trap to close. In *Mimosa*, the water pressure causes the pulvini to shrink, folding the leaflets together.

While it is important to acknowledge these physiological similarities in order to strengthen the comparison between the lineages under study, for the application of the present analysis we can blackbox most of these details. What is fundamental in the application of Chomsky’s hierarchy and automata theory to plant behaviour is the sensitivity of the plant to specific properties of the cue. Thus, assuming that *Aldrovanda* or *Drosera*, like *Dionaea* or *Mimosa*, are sensitive to ESs in a certain voltage range, we can think of a sequence of APs as generated by a grammar $\mathcal{G}$ with vocabulary Σ = {0, 1} as above, whose rules can be discovered by observing how the plant reacts to specific combinations. If a particular lineage displays responses corresponding to the acceptance of a particular language at some level of the subregular hierarchy, while another is apparently insensitive to the characteristic properties of that language, then we have *prima facie* reasons to say that the first lineage is more computationally complex than the second. Moreover, if we accept the view in formal language theory (Jäger and Rogers 2012; Fitch and Friederici 2012; Fitch 2014) that differences in computational complexity reveal different degrees of cognitive sophistication, then we have *prima facie* reasons to believe that the first lineage is more cognitively sophisticated than the second.

**Fig. 4:**
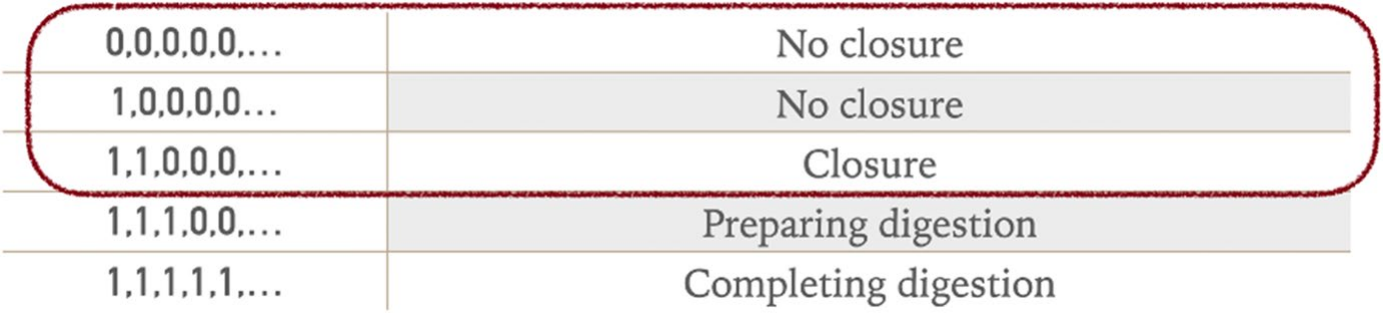
*Dionaea*’s sensitivity to specific sequences of APs

With this hypothesis in mind, let us first consider *Dionaea*, i.e., the Venus flytrap. As I mentioned in Sect. 2, the Venus flytrap has been extensively studied in the context of plant communication. In fact, along with other carnivorous plants, it has attracted the attention of biologists since Darwin’s time (Darwin 1880) because of its motility and reactivity. APs can be generated on the surface of *Dionaea*’s upper leaf epidermis by a variety of agents, especially touch. In particular, the plant’s epidermis contains hairs that are sensitive to the presence of insects or artificial stimuli. These cause the firing of individual potentials. Böhm et al. (2016) and Hedrich and Neher (2018) have investigated the details of *Dionaea*’s response to specific sequences of APs. One AP does not elicit any response. Two APs are sufficient to cause the closure of the trap. Three APs induce the production of a hormone called jasmonic acid, which activates the endocrine system and prepares the Venus flytrap for digestion. More than five APs amplify the activity of the flytrap endocrine system, favouring the complete digestion of the prey (assuming, of course, that the APs are caused by the presence of an insect rather than being artificially produced). Figure 4 reconstructs the sequence of APs to which *Dionaea* is sensitive in terms of the vocabulary Σ = {0, 1}.

The maximum time interval between the first and second AP is 30 s and the absence of further APs after the second can lead to an anticipated opening of the lobes (Hedrich and Neher 2018, p. 226). The number of APs required for trap closure can vary depending on external conditions (Hedrich and Neher 2018, p. 227) but, despite some variability, the overall behaviour is robust.

**Fig. 5:**
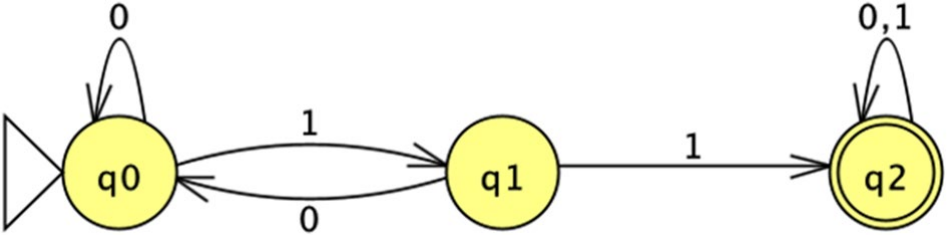
*Dionaea* automaton

Let us now look at the Venus flytrap as an automaton. To strengthen a possible comparison with plants such as *Mimosa pudica*, which is not carnivorous, I shall focus only on the stimulation/reaction steps (since *Mimosa*’s reactions serve a defensive role, it would be unfair to compare the two behaviours as a whole). The behaviour of the Venus flytrap can be modelled as a finite automaton as shown in Fig. 5 (I assume here, following Hendrich and Neher, that the lack of the second AP after 30 s resets the processing of the cue).

Symbolically, this automaton is defined by the tuple $<\left\{{q}_{o},{q}_{1},{q}_{2}\right\},\left\{\text{0,1}\right\}, \delta ,{q}_{0},{q}_{2}>$, where $\delta$ is the transition function defined in Table 1.

**Table 1:** *Dionaea* automaton’s transition function

	*0*	*1*
${q}_{o}$	${q}_{o}$	${q}_{1}$
${q}_{1}$	${q}_{0}$	${q}_{2}$
${q}_{2}$	${q}_{2}$	${q}_{2}$

The language accepted by this automaton is *L* $(D)=\{{0}^{*}1{1(0|1)}^{*}\}$, which is a simple LTT. This corroborates Hedrich and Neher’s hypothesis that the Venus flytrap has some capacity for *counting*. Using automata theory, this capacity can be more rigorously characterised as the ability to detect a threshold value for accepting (i.e., reacting to) a certain sequence of symbols/cues. No similar ability has been observed so far in *Mimosa pudica*, or in plants of the genera *Aldrovanda* and *Drosera*.

*Mimosa* is famous for its characteristic response to touch and vibration. When its leaflets are stimulated, *Mimosa* quickly closes them. The physiological details have been extensively studied, for instance, by Volkov et al. (2009). Apparently, the plant responds immediately after the first stimulation and shows no reactivity to further APs.^7^ It could be argued that the comparison between *Dionaea* and *Mimosa* is unfair because their reactions to APs serve different purposes. While *Dionaea*’s behaviour is related to resource gathering, *Mimosa*’s response is a protection against potentially harmful events (such as a herbivore bite). Note, however, that what is here at stake is not a functional analysis of the trait. It is certainly possible that certain environmental challenges favoured the evolution of more sophisticated responses. Nevertheless, what is being ascertained in the present context is not whether a behaviour is more or less adapted to a particular ecology. What is being inquired is the automata behaviour in similar mechanical circumstances. It may well be that *Mimosa* exhibits a more complex behaviour in different circumstances.^8^ However, in the particular case of APs processing for rapid responses, it displays a behaviour that is apparently simpler than *Dionaea*. In fact, it can be defined by the tuple $<\left\{{q}_{o},{q}_{1}\right\},\left\{\text{0,1}\right\}, \varepsilon ,{q}_{0},{q}_{1}>$, where $\varepsilon$ is the transition function defined in Table 2.

**Table 2:** *Mimosa* automaton’s transition function

	*0*	*1*
${q}_{o}$	${q}_{o}$	${q}_{1}$
${q}_{1}$	${q}_{1}$	${q}_{1}$

The language accepted by this automaton is *L* $(D)=\{{0}^{*}{1}^{+}\}$, where the symbol + denotes at least one occurrence of the symbol. *L* $(D)$ is an SL language, since neither quantification nor Boolean operations are required for the acceptance of a sequence of symbols. We can therefore conclude that *Mimosa* is a simple scanner, at least as far as this specific interaction is concerned.

There is less evidence about APs processing in the genera *Aldrovanda* or *Drosera*. This is the main reason why I focused on *M. pudica*, despite the functional differences between this plant and *Dionaea*. The available evidence on *Aldrovanda vesiculosa* (e.g., Iijima and Sibaoka 1981; Poppinga et al. 2019), an aquatic plant with a trap similar to that of a Venus flytrap, suggests that it is not capable of counting. The trap closes as soon as an AP is generated, revealing a simpler behavioural response to the cue that can be modelled similarly to the response of *M. pudica*. The case of *Drosera capensis* (as well of other plants of the genus *Drosera*) is potentially more interesting due to its distinctive morphological features (Ivesic et al. 2022). This plant has tentacles covered with an adhesive mucilage that respond to tactile stimulation. Under natural conditions, the presence of prey causes the tentacles to encircle and suffocate the prey. Due to the more flexible motility of the tentacles, *D. capensis* might appear to be able to display a more sophisticated behaviour. However, at least according to the analysis proposed here, the closure of the tentacles does not seem to show more computational complexity than that of *A. vesiculosa*. Nevertheless, Ivesic et al. (2022) provide evidence that the process of digestion could be sensitive to sequences of APs, similar to the flytrap’s case.

## Discussion

The above analysis is in many ways only a first, tentative step towards the application of automata theory to plant communication and behaviour (although it should be noted that a somewhat similar approach has already been proposed in the past by Kawano et al. 2012). The analysis is dependent on previous experimental protocols that, it goes without saying, were carried out without this specific application in mind. A more systematic and interdisciplinary effort should be made to classify the types of cues and the physiological mechanisms processing them, as well as to design specific experiments to compare the computational properties of these mechanisms. Despite its limitations, I believe that the work developed in the previous sections provides support for the following two considerations, as well as a motivation for experimenters to consider automata theory and the Chomsky hierarchy as valuable tools for future research.

Firstly, as mentioned above, the comparison between Venus flytrap and other species suggests that there are differences between them in the way they process artificially induced cues. These phenomena can be broadly described as communicative, in the sense that the plant’s behavioural flexibility can be exploited by researchers to elicit a range of desired responses in specific circumstances (see Sect. 2). In the context of comparative formal linguistics, behavioural flexibility is measured comparatively in terms of automata’s complexity. In turn, automata’s complexity is taken to reflect levels of cognitive sophistication. However, this inference from automata’s complexity to cognitive sophistication needs further clarification. Human beings, chimpanzees or birds, for instance, are able to communicate in certain ways *because* they have a brain, at least some capacity for abstract reasoning (e.g., Bugnyar et al. 2016) and characteristic desires. Thus, when we infer cognitive sophistication from communicative interactions in a particular species, we are taking behavioural evidence as an indication of differences between organisms that we already know to be cognitive agents, at least according to a largely shared common view (see, for instance, Lyon 2006; Allen and Trestman 2016; Adams 2017; Ginsburg and Jablonka 2019; Andrews and Monsó 2021).

In contrast, in the case of plant communication, there is no analogous consensually accepted evidence that a particular behaviour is due to cognitive features of the plant. Plants lack a nervous system (although some authors suggest that phloem and xylem might play an analogous role; Trewavas 2015; Calvo 2023), and there is no reason to believe they are able of some form of abstract reasoning or desire (at least not in a literal sense). Thus, when we talk about the “cognitive sophistication” of plants, we are somehow stretching the concept of cognition, so as to include in its characterisation some other features (possibly related to some more basic capacity for information processing; Thompson 2006). Concepts such as “cognition” or “communication” have long been subject to a process of “semantic pressure”, aimed at ridding them of unjustified anthropocentric, or even zoocentric, biases (Figdor 2018). According to Figdor, the successful extension of these concepts beyond their original domain requires a gradual and progressive consilience of experimental evidence, mathematical modelling and explanatory frameworks. I would thus argue that the inference from automata’s complexity to cognitive sophistication might be sound, as in the case of humans or animals, but only on the assumption that it is possible to provide additional *independent* reasons for thinking of plants as cognitive agents. Alone, automata’s complexity is insufficient to guarantee cognitive sophistication (otherwise every computer, or even a calculator, would be a cognitive agent).

Whether there are strong independent reasons to believe that plants are cognitive agents is not for me to decide in this article. However, it is important to note that, as argued by Colaço (2022; see also Akagi 2018), even if we ultimately reject the hypothesis that plants are cognitive agents, it may still be useful to model them *as if they were* cognitive agents (and the same point could be extended to computers, such as deep learning systems; see, for instance, Otsuka 2023, pp. 139–140). This is because, by stretching the most established conceptions of cognition through the analysis of borderline cases, we can learn more about cognition itself. In the present case, by analysing communication in animals and plants as analogous phenomena through the lens of automata theory, we can hope to elucidate similarities and differences in the mechanisms employed to generate specific responses. Whether we ultimately decide to label these mechanisms as cognitive in both animals and plants, or only in animals, we will have gained some insight into cognition.

Given these coordinates, *if* the supporters of plant cognition are right, and plant behaviour is the result of mechanisms that can be labelled as cognitive, *then* automata’s complexity can reveal different degrees of cognitive sophistication in plants. While this puts the burden of proof on the supporters of plant’s cognition (because they must provide independent reasons for the truth of the antecedent of the conditional), it also gives them some grounds for optimism. If different lineages display different levels of computational complexity– as in the case of Venus compared to *Mimosa*, *Aldrovanda* and *Drosera* in our case study–then it is natural to wonder about the causes of these different behaviours. While there is no guarantee that Venus’ ability to count is the result of cognitive processes, the fact that Venus displays such an ability (as evidenced by the fact that we need an LTT acceptor to model its behaviour) at least *encourages* a cognitive explanation of its behaviour. This is because counting (albeit to the limited extent allowed by an LTT grammar; see Sect. 3) usually involves the presence of some–at least rudimentary–memory, which in turn is considered an important criterion for cognition (e.g., Adams 2017; Ginsburg and Jablonka 2019). On the other hand, if we ultimately reject the hypothesis that plants are cognitive agents on independent grounds, this evidence should stimulate further research into how it is possible for an organism without cognitive capacities is able to function as an automaton at a particular level of Chomsky’s hierarchy.

Focusing on the identification of abilities related to a particular level of Chomsky’s (or, more precisely, subregular) hierarchy–such as the ability to perform Boolean operations and basic counting–is, I believe, a promising perspective for the field of plant cognition. What is perhaps less promising is the fact that even the supposedly most sophisticated of the plants considered above–i.e., Venus flytrap (or, possibly, *D. capensis*, if Ivesic et al. 2022’s findings are corroborated)–does not seem to score very well from the point of view of Chomsky’s hierarchy. Although they can perform Boolean operations and basic counting, LTT acceptors such as Venus flytrap in our present case study are unable to perform other simple operations, such as those requiring comparisons involving the order of elements (Jäger and Rogers 2012, p. 1965). This ability, displayed by automata above the LTT level, can arguably be related to a primitive capacity for prediction, since an automaton capable of recognising ordered patterns can be cognitively characterised as *expecting* certain sequences of signals, while rejecting those that do not match the expected patterns.^9^ The fact that none of the specimens analysed in Sect. 4 show behaviour above the LTT level could be taken as evidence that these behaviours do not involve capacities of prediction, contrary to what some advocates of plant cognition might believe.

Remember from the introduction that the main aim of this article was to compare plant cognition with that of humans and animals. If we rely on the proposed comparative measure, then we can say that the ability of plants to respond to induced stimuli is quite limited. This is perhaps not surprising, but it suggests that a more cautious approach to plant cognition should be adopted. Of course, new evidence could overturn this tentative conclusion. For example, if we were able to identify plant’s reactions to APs with voltages in different ranges (rather than in a single definite range, as in the cases analysed above), we could interpret them as sensitive to a more complex vocabulary (because each range could be digitalised as a different symbol). The experimenters could thus produce more complex sequences of activations in order to elicit more sophisticated responses, perhaps displaying a behaviour corresponding to more complex automata in the hierarchy of automata. Moreover, research on other types of stimuli, displaying more variation–such as AVs–or combinatorial properties–such as VOCs–might help to broaden our view on the communicative abilities of plants and, possibly, about their cognitive sophistication.

## Conclusion

In this article I proposed a possible application of Chomsky’s hierarchy and automata theory to model plant behaviour. In doing so, I have focused on what is called “plant communication”, which includes both plant-plant communicative interactions and artificial stimulation aimed at eliciting specific responses. For reasons of simplicity and the lack of more experimental evidence, I have focused mainly on the latter. After discussing the link between Chomsky’s hierarchy, automata theory and cognitive sophistication from the perspective of comparative formal linguistics in Sect. 3, I have analysed automata’s complexity of some plant behaviours in different lineages in Sect. 4. These lineages–i.e., *Dionaea muscipula*, *Mimosa pudica* and plants belonging to the genera *Aldrovanda* and *Drosera*–exhibit rapid movements in response to electrical stimulation. From the application of automata theory to behaviours resulting from similar physiological mechanisms, it can be extrapolated that *Dionaea muscipula* has a certain capacity to perform Boolean operations (related to the discrimination of specific sequences of symbols) and counting. This result, although tentative, suggests two things. Firstly, it shows that different lineages, under similar mechanical conditions of activation, can react in more or less complex ways. This may lead to the speculation that they have different degrees of cognitive capacities. Although the tenability of this conclusion depends heavily on the definition of cognition used, it encourages further comparative studies. Secondly, the case studies analysed also show the limits of the communicative behaviours of plants. In particular, if we accept Jäger and Rogers’ (2012) cognitive characterisation of automata accepting subregular languages, none of the discussed lineages is apparently able to recognise order relations in sequences of activation. This might suggest a limited capacity of prediction.

The proposed analysis is admittedly only a first attempt to bring formal tools to the field of plant cognition. While further experimental protocols explicitly designed to test the assumptions involved in the application of automata theory would help to make this methodology more rigorous, the present work has argued that these applications can provide useful insights into the cognitive abilities of plants.

## Data Availability

This paper utilizes data and findings from published research articles. All relevant sources are fully cited within the text and listed in the References section. Due to the philosophical nature of the inquiry, no original data collection was conducted for this paper.

## Footnotes

1 Of course, this does not mean that selection does not play a role in eavesdropping. Acoustic sensitivity to insect chewing (Appel and Cocroft 2014), photoreception of shading (Novoplansky et al. 1990) or touch sensitivity (Pavlovic et al. 2017) are all arguably adaptive behaviours in which unintentional cues are exploited to increase survival chances.

2 The mathematical proof that a given language cannot be generated from a given grammar is usually based on what is called the “pumping lemma” for that particular grammar. Since these technical details are not relevant here, the interested reader can consult Hopcroft et al. (2008) or Sipser (2020) for rigorous discussions of the pumping lemma for regular and context-free grammars.

3 The name “dendrophilia” comes from ancient Greek δένδρον (“tree”) + φίλια (“love”). Fitch’s hypothesis is so called because context-free embeddings are often represented by tree-like graphs. Thus, “dendrophilia” refers to our particular affinity for context-free linguistic structure, which, according to Fitch, is absent in other animals (or, more generally, living beings).

4 This is the case, for instance, when we input strings of the language *L* $({\mathcal{G}}_{1})=\{{\left(ab\right)}^{n}\bullet {\left(cd\right)}^{n}|n \in {\mathbb{N}}^{+}\}$ into a finite automaton designed to accept the regular language *L* $({\mathcal{G}}_{3})=\{{\left(ab\right)}^{3}\bullet {\left(cd\right)}^{3}\}$. The automaton will accidentally accept strings of the form ${\left(ab\right)}^{3k}\bullet {\left(cd\right)}^{3k}, k \in {\mathbb{N}}^{+}$, although it is not designed to do so. This is again a consequence of the pumping lemma mentioned in footnote 2.

5 To express regular languages, instead of using the context-free grammar formalism employed at the beginning of this session, it is more practical to use the regular expressions formalism here adopted.

6 Note that the *L* $({LTT}_{1})$ acceptor does not process, if not incidentally, strings containing *exactly* three *ab* sequences. This is because, after scanning the third *ab* sequence, it enters an accepting state that is insensitive to any further input. However, other LTT acceptors could be designed to accept sequences containing a block of fixed length.

7 For a nice illustration of the different sensitivities of *Dionaea* and *Mimosa* see neuroscientist Greg Gage’s TED talk (available at https://www.youtube.com/watch?v=pvBlSFVmoaw).

8 Gagliano et al. (2014) refer to an experiment in which *Mimosa* is exposed to repeated falls, to which it initially reacts as expected (by closing the leaflets). However, Gagliano and her collaborators claim that, after a certain number of falls, the plant is able to habituate to the stimulus and recognise that it does not pose a danger, thus keeping the leaflets open after a fall. Furthermore, Gagliano et al. argue that the plant is able to remember what it has learned about the stimulus up to a month after the initial habituation. As far as I know, no researcher has yet been able to replicate Gagliano et al.’s experiment. In any case, there are two things to note. Firstly, if the experiment were to be replicated with the same result, it would certainly be interesting to further study the biochemical mechanisms responsible for the behaviour and to interpret them through the lens of automata theory in order to explore *Mimosa*’s cognitive sophistication in more detail. This means that the correctness of Gagliano et al.’s results would not invalidate the approach proposed here. Secondly, even if *Mimosa* is capable of more sophisticated cognitive abilities than those discussed in the text, this is not incompatible with the fact that, *in the specific circumstances* being analysed, its behaviour is simpler than that of the flytrap.

9 An example of such an automaton is the automaton which, given Σ = {0, 1}, accepts a language *L* $=\{0011{(0|1)}^{*}\}$, according to which a sequence of two zeros must precede a sequence of two ones before any other symbol occurs.
